# Satellite Cell Isolation, Culture, and Infection After Retroviral Preparation

**DOI:** 10.21769/BioProtoc.5751

**Published:** 2026-07-20

**Authors:** Chuanli Zhou, Yue Lu, Elizabeth H. Chen

**Affiliations:** 1 Department of Biochemistry and Molecular Biology, University of Iowa, Iowa City, IA, USA; 2 Department of Molecular Biology, University of Texas Southwestern Medical Center, Dallas, TX, USA; 3 Department of Cell Biology, University of Texas Southwestern Medical Center, Dallas, TX, USA; 4 Hamon Center for Regenerative Science and Medicine, University of Texas Southwestern Medical Center, Dallas, TX, USA; 5 Harold C. Simmons Comprehensive Cancer Center, University of Texas Southwestern Medical Center, Dallas, TX, USA

**Keywords:** Skeletal muscle, Satellite cell, Retroviral transduction, Genetic manipulation, Cell culture

## Abstract

Satellite cells are adult skeletal muscle stem cells that play essential roles in muscle regeneration. Understanding their behavior is critical for elucidating the mechanisms of muscle repair and advancing muscle regenerative therapies. This requires efficient methods for genetic manipulation in these cells. Retroviral-mediated gene delivery is commonly used for stable transgene expression in immortalized cell lines. However, existing approaches are not optimized for primary satellite cells, often resulting in variable efficiency and inconsistent outcomes. Here, we describe an optimized protocol for satellite cell isolation and culture, as well as retroviral production and infection of primary satellite cells that achieves high transduction efficiency. The satellite cell isolation procedure enriches for myofiber fragments prior to satellite cell release, thereby reducing contamination by non-myogenic cells and improving cell purity. Another key feature of this protocol is the concentration of retroviral particles and their resuspension in satellite cell growth medium prior to infection, which minimizes satellite cell exposure to packaging cell-conditioned medium. Compared to standard approaches, this protocol improves both infection efficiency and reproducibility. It is readily adaptable to a wide range of downstream applications, including microscopies, biochemical assays, and molecular biology analyses.

Key features

• Optimization of culture conditions for satellite cells, ensuring high retroviral transduction efficiency.

• Efficient production and concentration of retroviral particles for reliable infection of satellite cells.

• Compatibility with multiple downstream applications for studying satellite cell biology, including microscopies, biochemical assays, and molecular biology analyses.

## Graphical overview


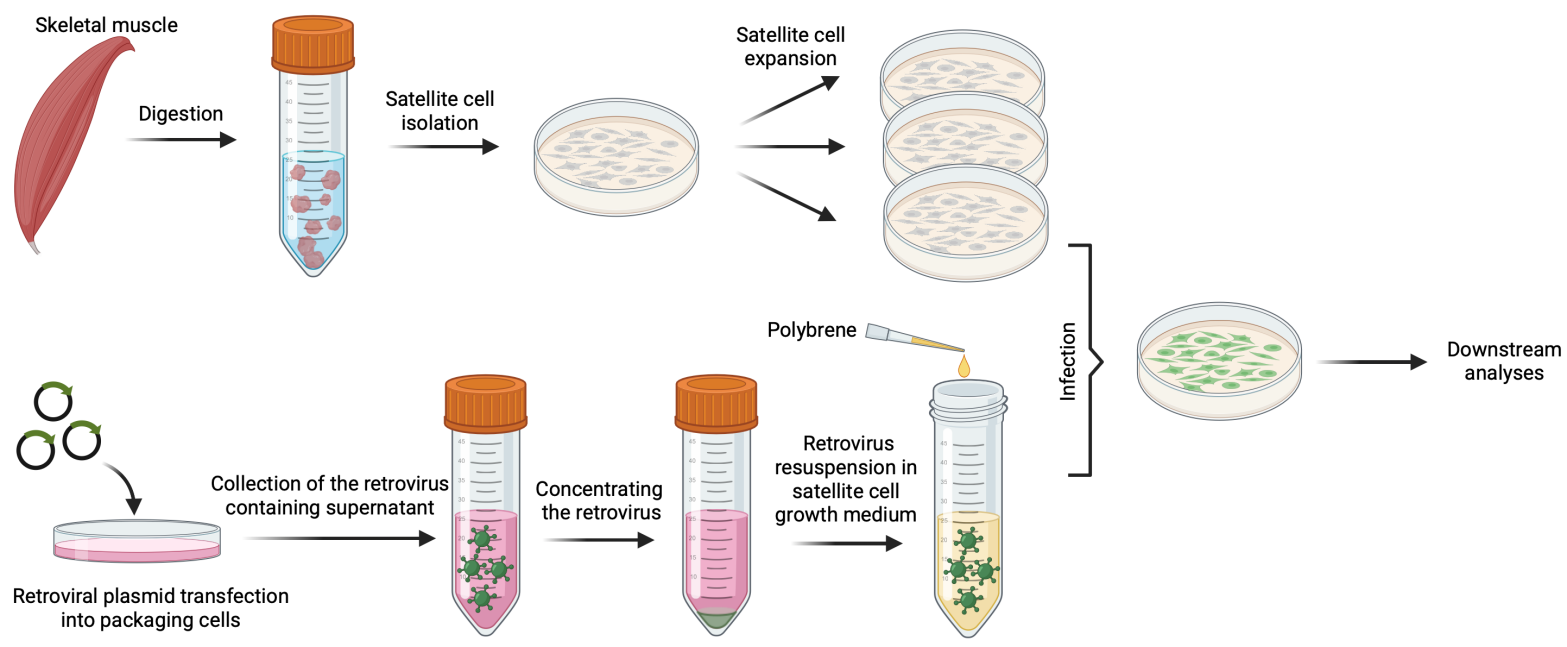


**Workflow for mouse satellite cell isolation, culture, and retroviral transduction**

## Background

Satellite cells are adult skeletal muscle stem cells that play essential roles in muscle regeneration [1–5]. Upon skeletal muscle injury, satellite cells exit quiescence, proliferate, differentiate, and fuse to regenerate the damaged muscle fibers [1,4–10]. Understanding the molecular mechanisms that regulate satellite cell behavior requires efficient and reliable methods for genetic manipulation in these cells. Although several methods for satellite cell isolation have been reported across multiple species [11–19], protocols for reliable genetic perturbation in these primary cells remain less well established.

Retroviral-mediated gene delivery is a widely used approach for stable expression of transgenes in cells [20,21]. Compared to plasmid transfection, retroviral transduction typically yields higher efficiency and more uniform gene expression in cells [22]. In primary cells, retroviral infection methods are generally adapted from protocols developed for immortalized cell lines [23–26]. However, these approaches are not specifically optimized for satellite cells, which are highly sensitive to culture conditions, such as medium composition and serum levels. Components present in viral supernatant may alter the culture environment, leading to reduced proliferation and enhanced differentiation, which in turn limits retroviral integration. As a result, these methods often result in variable infection efficiency and inconsistent outcomes in primary satellite cells.

In this protocol, we describe an optimized method for retroviral production and infection of primary satellite cells. Compared to standard retroviral approaches, this protocol concentrates retrovirus produced in HEK293 cell-conditioned medium and resuspends it directly into satellite cell growth medium prior to infection. This strategy minimizes the exposure of satellite cells to HEK293 cell-conditioned medium, which can adversely affect satellite cell proliferation and promote premature differentiation, while maintaining high infection efficiency and reducing experimental variability. It is compatible with a wide range of downstream applications, including microscopies, biochemical assays, and molecular biology analyses. This method can be broadly applied to study satellite cell proliferation, differentiation, fusion, and gene function in skeletal muscle biology.

## Materials and reagents

**Biological materials**

1. 2-month-old wild-type C57BL/6J male mice (Jackson Laboratory, stock: 000664)

2. Platinum-E cells (Cell Biolabs, catalog number: RV-101)

**Reagents**

1. Retroviral vector pMXs-LifeAct-mNeongreen; described in a previous study [27]

2. FuGENE HD transfection reagent (Promega, catalog number: E2311)

3. Acetic acid (Sigma, catalog number: A6283-500ML)

4. Type II collagenase (Worthington, catalog number: LS004176)

5. Dispase (Gibco, catalog number: 17105041)

6. Horse serum (Gibco, catalog number: 16050122)

7. F-10 Ham’s medium (Thermo Fisher Scientific, catalog number: 11550043)

8. DMEM (Thermo Fisher Scientific, catalog number: 11965092)

9. FBS (Thermo Fisher Scientific, catalog number: A5670701)

10. Ethanol, 140 proof (Fisher Scientific, catalog number: 04-355-309)

11. FGF2 (Thermo Fisher Scientific, catalog number: 100-18B-50UG)

12. Penicillin-streptomycin (Thermo Fisher Scientific, catalog number: 15140122)

13. HEPES (1M) (Thermo Fisher Scientific, catalog number: 15630080)

14. Collagen I, rat tail (Gibco, catalog number: A1048301)

15. PBS (1*×*), pH 7.4 (Fisher Scientific, catalog number: 10010072)

16. Opti-MEM^TM^ reduced serum medium (Fisher Scientific, catalog number: 31985062)

17. Retro-X^TM^ concentrator (TaKaRa, catalog number: 631456)

18. Trypsin-EDTA (0.25%) (Thermo Fisher Scientific, catalog number: 25200072)

19. DMSO (Sigma, catalog number: D4540)

20. Polybrene, 10 mg/mL (Sigma, catalog number: TR-1003-G)

**Solutions**

1. 0.02 M acetic acid (see Recipes)

2. Coating buffer (see Recipes)

3. Wash medium (WM) (see Recipes)

4. Digest solution 1 (DS1) (see Recipes)

5. Digest solution 2 (DS2) (see Recipes)

6. Growth medium (GM) (see Recipes)

7. Platinum-E cell growth medium (PEGM) (see Recipes)

**Recipes**

*Note: All homemade solutions are sterilized by filtration through a 0.22 μm PES membrane.*

**1. 0.02 M acetic acid**

**Table:** 

Reagent	Final concentration	Quantity or volume
17.4 M acetic acid	0.02 M	1 mL
Sterile distilled water		Up to 870 mL

Store at room temperature for up to 1 year.

**2. Coating buffer**

**Table:** 

Reagent	Final concentration	Quantity or volume
Collagen I (3 mg/mL)	50 μg/mL	833 μL
0.02 M acetic acid		Up to 50 mL

Prepare fresh before use.

**3. Wash medium (WM)**

**Table:** 

Reagent	Final concentration	Quantity or volume
Penicillin-streptomycin (100×)	1%	5 mL
Horse serum	10%	50 mL
F-10 Ham’s medium		Up to 500 mL

Prepare fresh before use.

**4. Digest solution 1 (DS1)**

**Table:** 

Reagent	Final concentration	Quantity or volume
Type II collagenase	800 U/mL	The amount of powder is batch-specific
Wash medium		100 mL

Prepare fresh before use.

**5. Digest solution 2 (DS2)**

**Table:** 

Reagent	Final concentration	Quantity or volume
Dispase	0.5 U/mL	The amount of powder is batch specific
Type II collagenase	40 U/mL	The amount of powder is batch specific
Penicillin–streptomycin	1%	0.1 mL
Horse serum	10%	1 mL
F-10 Ham’s medium		Up to 10 mL

Prepare fresh before use.

**6. Growth medium (GM)**

**Table:** 

Reagent	Final concentration	Quantity or volume
FBS	20%	100 mL
FGF2	4 ng/mL	25 μL (80 μg/mL)
Penicillin-streptomycin	1%	5 mL
HEPES	1%	5 mL
F-10 Ham’s medium		Up to 500 mL

Store at 4 °C for up to one month. Stock FGF2 (80 μg/mL) can be stored at -20 °C for up to a year.

**7. Platinum-E cell growth medium (PEGM)**

**Table:** 

Reagent	Final concentration	Quantity or volume
FBS	10%	50mL
Penicillin-streptomycin	1%	5 mL
HEPES	1%	5mL
DMEM		Up to 500 mL

Store at 4 °C for up to 1 month.

**Laboratory supplies**

1. 50 mL Falcon tubes (Fisher Scientific, catalog number: 14-432-22)

2. 15 mL Falcon tubes (Fisher Scientific, catalog number: 14-959-53A)

3. 10 cm TC-treated cell culture dish (Corning, catalog number: 353003)

4. 35 mm TC-treated cell culture dish (Corning, catalog number: 430165)

5. Syringe filter, SFCA, 0.45 μm (Santa Cruz, catalog number: sc-516782)

6. Sterile cell strainers (Fisher Scientific, catalog numbers: 07-201-430, 07-201-432)

7. 500 mL vacuum filter/storage bottle system (Corning, catalog number: 431097)

8. 150 mL vacuum filter/storage bottle system (Corning, catalog number: 431153)

9. Syringe filter, SFCA, 0.22 μm (Santa Cruz, catalog number: SC-516781)

10. 10 mL syringe (Santa Cruz, catalog number: SC-358907)

11. Sterile disposable serological pipettes (Fisher, catalog numbers: 1367811D, 1367811E)

12. 0.22 μm PES membrane (Sigma, catalog number: GPWP04700)

## Equipment

1. Dissecting tissue forceps (Fisher Scientific, catalog number: 13-812-41)

2. Sharp-pointed dissecting scissors (Fisher Scientific, catalog number: 08-940)

3. Motorized pipette controller (Eppendorf, catalog number: 4430000018)

4. Nutating mixer (Fisher Scientific, catalog number: 88-861-041)

5. Benchtop centrifuge (Eppendorf, model: 5810R)

6. CoolCell LX cell freezing container (Corning, catalog number: CLS432002)

7. Sterile hood (Thermo Fisher Scientific, model: 1500 Series A2 Class II)

8. Water bath (Thermo Fisher Scientific, catalog number: TSGP10)

9. CO_2_ incubator (PHCbi, model: MCO-230AICUVLG-PA)

## Software and datasets

1. Fiji software (ImageJ2 distribution), v2.14.0 (released 2023/11/15) [34]. Free, open source (GPL license). Used for image processing, background subtraction, drift correction, and kymograph generation. Download at https://imagej.net/software/fiji/downloads

## Procedure

**A. Satellite cell isolation and culture**

*Note: This method first isolates myofibers and subsequently releases satellite cells from these fibers. This approach enriches for satellite cells and reduces contamination by fibroblasts and other non-myogenic cells, thereby improving cell purity. Depending on the experimental conditions and isolation efficiency, this enrichment strategy may result in a lower overall cell yield than direct mononuclear cell isolation methods.*

**A1. Prepare collagen-coated dishes**

*Note: Prepare dishes the day before dissection.*

1. Make the coating buffer fresh and add 4 mL of coating buffer to each 10-cm TC dish, or 1 mL of coating buffer to each 35-mm dish to coat the surface.

2. Incubate the dishes with coating buffer at 37 °C for 1 h.

3. Aspirate off the coating buffer, wash the dishes with 1× PBS three times, and place the dishes with lids on in a tissue culture hood until dry.

*Note: The coated dry dishes can be stored at room temperature for up to 1 month.*

**Figure 1.**
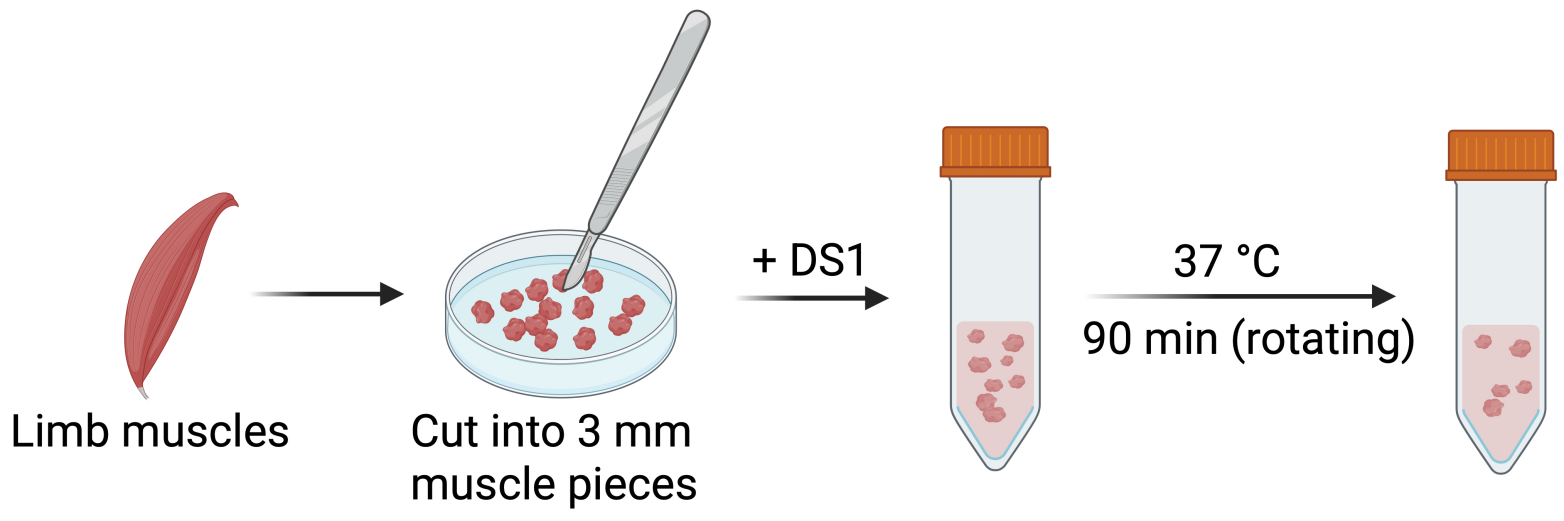
Limb muscle dissection and muscle fiber dissociation

**A2. Dissect muscle and dissociate muscle fibers** (Figure 1)

*Note on animal ethics: The use of animals was approved by the University of Iowa Animal Care and Use Committee according to NIH guidelines.*

1. Euthanize mice by CO_2_ asphyxiation followed by cervical dislocation. Spray the mouse liberally with 70% ethanol and place it on its back in the dissection area.

2. De-skin the legs and remove muscles from the hindlimbs and forelimbs. Collect muscles in a 10-cm TC dish containing 10 mL of 1× PBS. Remove visible fat, bone, and tendon.

3. Move isolated muscle tissue to a new Petri dish containing 10 mL of 1× PBS.

4. Remove PBS with a serological pipette. Cut the muscle into approximately 3-mm pieces.

**Critical:** Do not mince the tissue finely.

5. Use a ratio of 1 g of muscle for 3 mL of DS1.

*Note: DS1 should be prepared freshly by dissolving collagenase II powder in WM before the dissection and keeping it on ice until needed.*

6. Transfer chopped tissue to a 50 mL conical tube and incubate at 37 °C for 90 min on a nutating mixer (20–25 rpm). **Critical:** Perform all subsequent manipulations in a tissue culture hood.

**Figure 2.**
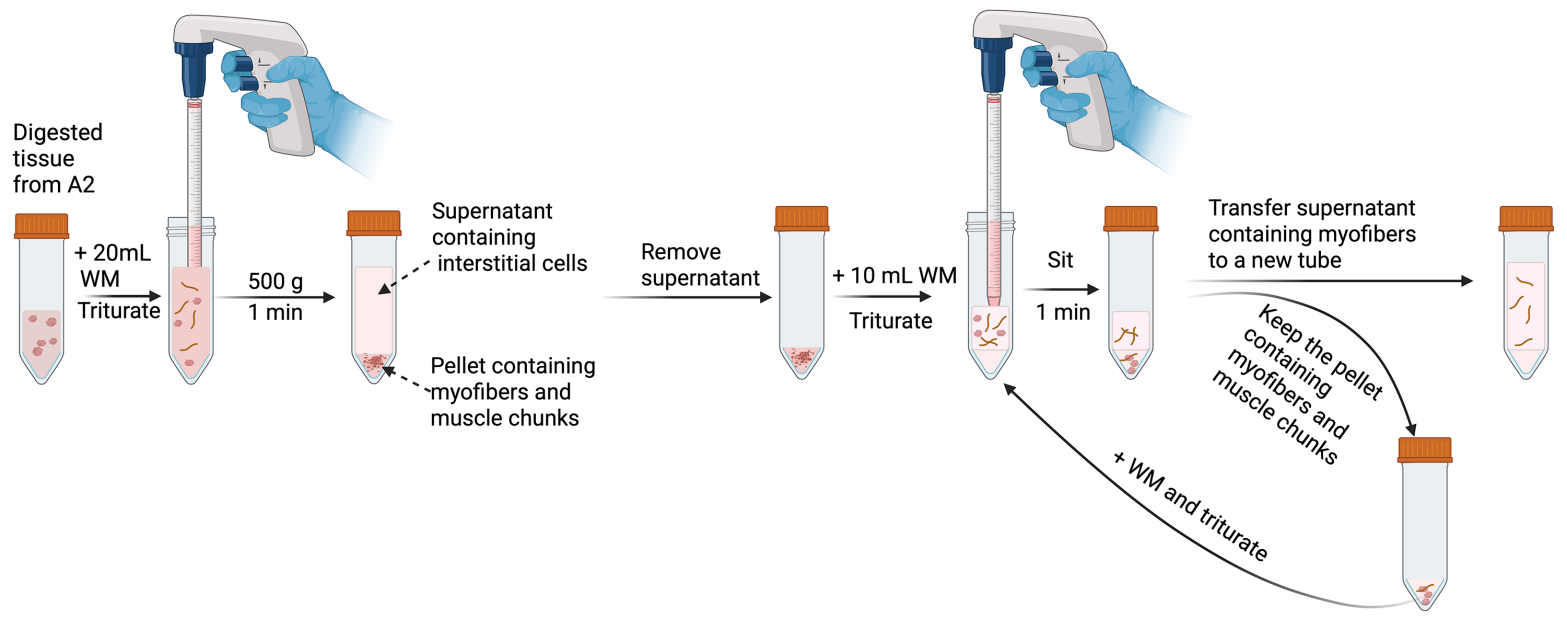
Enrichment of single myofibers

**A3. Enrich single myofibers** (Figure 2)

1. After the 90-min digestion, fill the tube to 20–30 mL with GM to quench digestion.

2. Triturate repeatedly with a serological pipette to dissociate muscle chunks into single myofibers.

3. Centrifuge at 500× *g* for 1 min to pellet myofibers and muscle chunks.

4. Carefully remove the supernatant with a serological pipette. This supernatant contains interstitial cells, mostly fibroblasts.

*Note: The pellet is loose and can be easily disturbed.*

5. Add 10 mL of WM to the tube containing the pellet. Triturate five times to disperse the single fibers. Let the tube sit for 1 min at room temperature so the large chunks settle to the bottom.

6. Carefully transfer the supernatant containing single-fiber fragments into a new 50 mL tube without disturbing the chunk at the bottom.

7. Repeat this wash/transfer step two more times. Pool all collected supernatants in a 50 mL conical tube.

**Figure 3.**
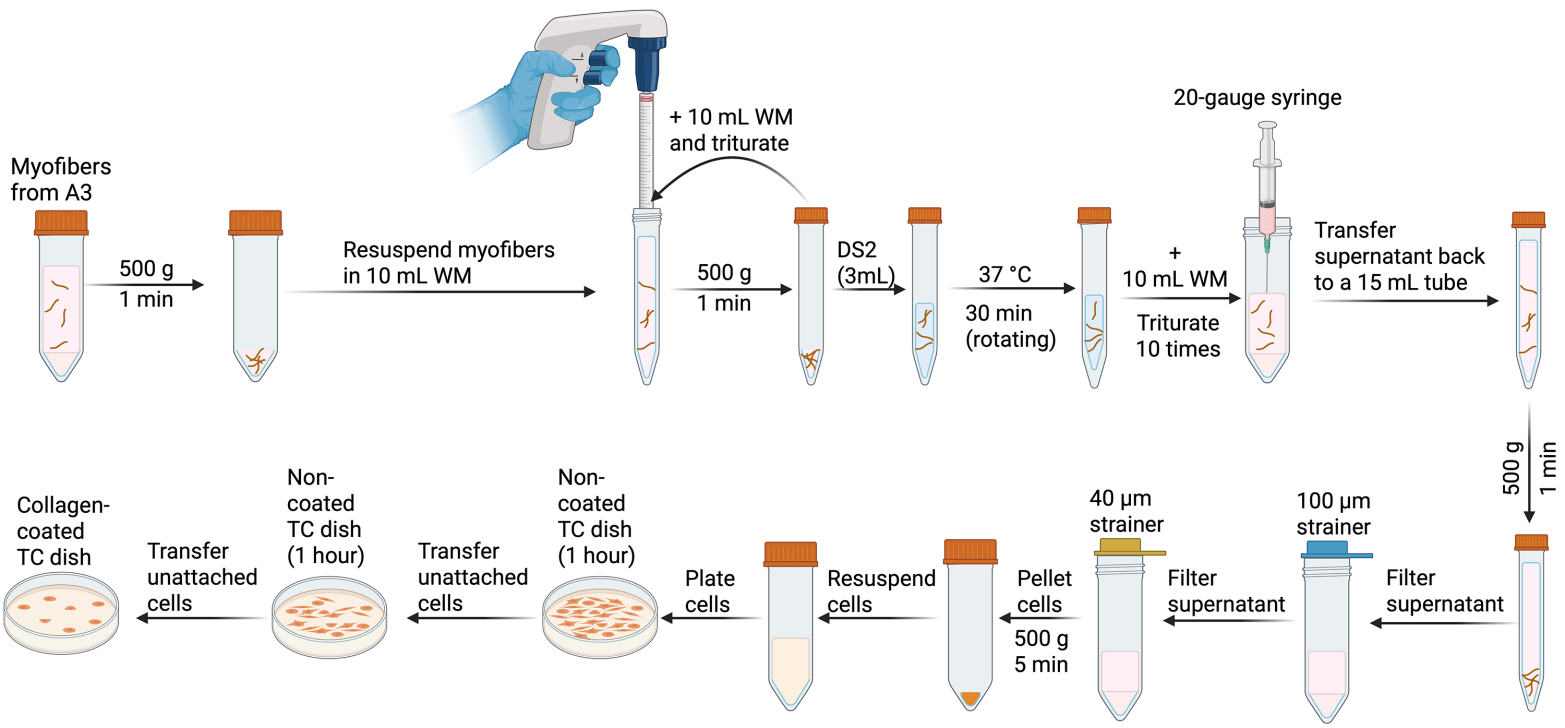
Release satellite cells from myofibers and enrich satellite cells by pre-plating

**A4. Release and enrich satellite cells** (Figure 3)

1. Centrifuge the tubes at 500× g for 1 min. Discard the supernatant.

2. Add 10 mL of WM. Transfer the single fiber containing WM to a 15 mL conical tube. Triturate five times to disperse the single fibers. Centrifuge the tubes at 500× g for 1 min. Discard the supernatant.

3. Add 10 mL of WM and repeat the wash two more times, leaving a pure population of myofibers and their associated mononucleated cells.

4. Add 3 mL of DS2 to each tube containing the myofiber pellet. Incubate at 37 °C for 30 min on a nutating mixer (20–25 rpm).

**Critical:** This step is time sensitive. Do not digest for longer than 40 min.

5. Add 10 mL of WM to the digested mixture and gently mix well.

6. Transfer the solution with myofibers to a 50-mL conical tube. Liberate satellite cells from the myofibers by triturating the mixture 10 times with a 20-gauge syringe.

7. After trituration, transfer the solution back to a 15 mL conical tube.

8. Centrifuge the tube at 500× *g* for 1 min to pellet fiber debris.

9. Filter the supernatant containing satellite cells sequentially through 100 and 40 μm cell strainers.

10. Collect the flowthrough in a 50 mL conical tube. Centrifuge at 500× *g* for 5 min to pellet satellite cells. Aspirate the supernatant.

11. Gently resuspend the pellet in 12 mL of GM.

12. Plate the mononucleated cells on a non-coated 10-cm TC dish. Incubate the cells in the incubator at 37 °C with 5% CO_2_ for 1 h.

*Note: This step enriches satellite cells by exploiting the fact that fibroblasts adhere faster to uncoated plastic than satellite cells. Fibroblasts preferentially attach, and satellite cells remain in suspension. This step significantly reduces fibroblast contamination.*

13. After 1 h, transfer the supernatant with the unattached cells to a new non-coated 10-cm TC dish. Incubate cells in the incubator at 37 °C with 5% CO_2_ for 1 h. After another hour, repeat step A4.13 one more time. Then, plate the supernatant with the unattached cells to the collagen-coated 10-cm TC dish.

*Note: A typical isolation from the pooled forelimb and hindlimb muscles of a 2-month-old wild-type C57BL/6J male mouse yields approximately 2–4 × 10^4^ satellite cells. The exact number may vary depending on factors such as mouse age, sex, muscle mass, and isolation efficiency.*

**A5. Satellite cell culture and maintenance**

1. Culture plated cells undisturbed. After 48 h, change to fresh GM.

*Note: Do not change medium during the first 2 days, to allow cells to adhere to the substrate.*

**Figure 4.**
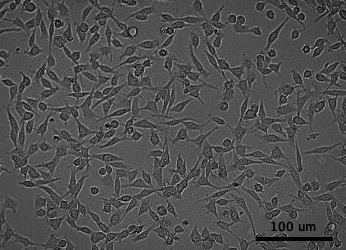
Representative image of 60% confluent satellite cells

2. Once the cells reach 60% confluency (Figure 4), aspirate the medium, gently wash the cells once with 1× PBS, and digest with 2 mL of Trypsin-EDTA (0.25%) at 37 °C for 1 min.

*Note: Monitor under the microscope until the cells round up and detach.*

3. Stop the digestion by adding 2 mL of GM. Pipette gently up and down to obtain a single-cell suspension.

4. Transfer the cell suspension to a conical tube. Centrifuge at 300× *g* for 2 min.

5. Aspirate the supernatant. Resuspend the pellet in fresh GM.

6. Split cells at a 1:3 ratio (~1 × 10^4^ cells/cm^2^) for routine expansion.

7. After passaging, return cells to the incubator at 37 °C with 5% CO_2_. Change medium the next day if needed to remove dead cells and debris.

8. Cryopreservation: The trypsinized satellite cells in step A5.5 can be cryopreserved in GM containing 10% DMSO. Place the cryovials in a cell freezing container at -80 °C overnight, then transfer the vials to liquid nitrogen for long-term storage.

9. Thawing: Quickly thaw the frozen vial containing satellite cells in a 37 °C water bath. Transfer the cell suspension into a 15 mL conical tube containing 9 mL of GM. Centrifuge at 300× *g* for 2 min.

10. Resuspend the pellet in 10 mL of GM and transfer the suspension into a collagen-coated 10-cm TC dish.

**Figure 5.**
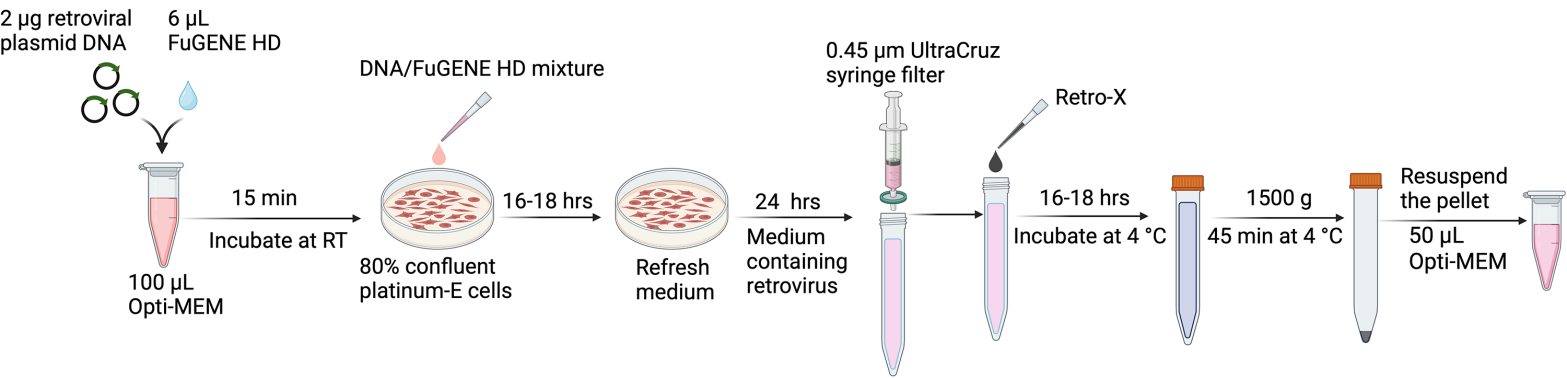
Retrovirus packaging and concentration

**B. Retroviral particle preparation** (Figure 5)

*Note: This protocol is based on retrovirus preparation for infection of satellite cells in a single 35-mm TC dish. Reagent volumes should be scaled proportionally as needed when processing additional wells or dishes. In addition, all procedures involving retroviral production and infection should be performed in accordance with institutional biosafety regulations and approved biosafety protocols, typically under BSL-2 containment.*

**Figure 6.**
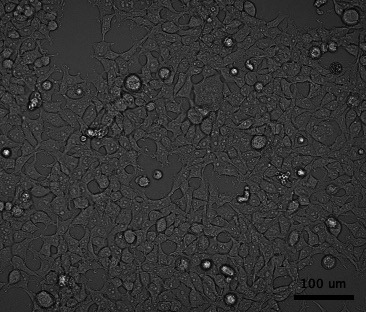
Representative image of 80% confluent platinum-E cells

1. One day before transfection, plate platinum-E cells in a 35-mm TC dish at 80% confluency (~1 × 10^5^ cells/cm^2^) in 2 mL of PEGM (Figure 6).

2. After 24 h, add 2 μg of retroviral plasmid DNA (e.g., pMx-LifeAct-mNeonGreen) into 100 μL of Opti-MEM reduced serum medium in a 1.5 mL centrifuge tube. Pipette to mix the plasmid with the medium gently.

3. Add 6 μL of FuGENE HD transfection reagent to the Opti-MEM medium containing plasmid DNA and mix immediately.

*Notes:*

*1. Before starting, allow the Opti-MEM reduced serum medium and FuGENE HD transfection reagent to reach room temperature.*

*2. Minimize the contact of undiluted FuGENE HD reagent with plastic surfaces, as chemical residues in plastic can significantly decrease the biological activity of the reagent. Always dilute the reagent by pipetting directly into the medium. Do not allow the FuGENE HD reagent to contact the plastic walls of the tube containing the medium during the dilution step.*

4. Incubate the FuGENE HD transfection reagent/DNA mixture for 15 min at room temperature.

5. Add the FuGENE HD transfection reagent/DNA mixture to the 35-mm TC dish containing platinum-E cells in 2 mL of PEGM. Mix by gently shaking the plate. Return cells to the incubator.

6. After 16–18 h, replace the medium with fresh PEGM.

7. After another 24 h, collect the culture medium containing the retrovirus and filter the medium using the 0.45 μm UltraCruz syringe filter to remove any detached Platinum-E cells and large cellular debris.

**Critical:** Use only cellulose acetate or polyethersulfone (PES) (low protein binding) filters. Do not use nitrocellulose filters. Nitrocellulose binds surface proteins on the retroviral envelope and destroys the packaged virus.

8. Transfer the clarified medium to a 15 mL conical tube and combine 1 volume of Retro-X concentrator with 3 volumes of clarified medium. Mix by gentle inversion.

9. Incubate the mixture overnight at 4 °C. Then, centrifuge the sample at 1,500× *g* for 45 min at 4 °C. Carefully remove the supernatant, taking care not to disturb the pellet.

10. Gently resuspend the pellet in 50 μL of Opti-MEM reduced serum medium.

11. Immediately use the concentrated virus to infect satellite cells or store at -70 °C for future use.

*Note: To minimize loss of infectivity, aliquot viral preparations before storage and avoid repeated freeze–thaw cycles.*

**C. Satellite cell infection after retroviral particle preparation**

*Note: This protocol is based on the infection of satellite cells in a single 35-mm TC dish. Reagent volumes should be scaled proportionally when processing additional wells or dishes.*

**Critical:** Retroviral particles should be concentrated from the Platinum-E-conditioned medium and resuspended directly in satellite cell GM before infection. Prolonged exposure to Platinum-E-conditioned medium can negatively affect satellite cell proliferation and promote premature differentiation. This strategy minimizes such effects while maintaining high infection efficiency and improving experimental consistency.

**Figure 7.**
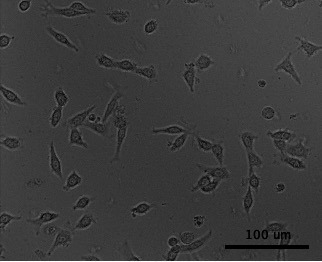
Representative image of 30% confluent satellite cells

1. One day before infection, plate the satellite cells in a collagen-coated 35-mm TC dish at 30% confluency (1.5 × 10^4^ cells/cm^2^) in 2 mL of GM (Figure 7).

**Critical:** Satellite cells must be maintained in an actively proliferating state prior to plating for infection, as retroviral infection is restricted to infecting dividing cells.

2. Add 50 μL of concentrated retroviruses into 2 mL of warm GM, then add 1.4 μL of polybrene (10 mg/mL). Mix by pipetting gently.

*Note: Viral titers are not routinely determined, but the amount of concentrated retrovirus used in this protocol was optimized for cells in a 35-mm TC dish and routinely yields efficient infection of satellite cells.*

**Figure 8.**
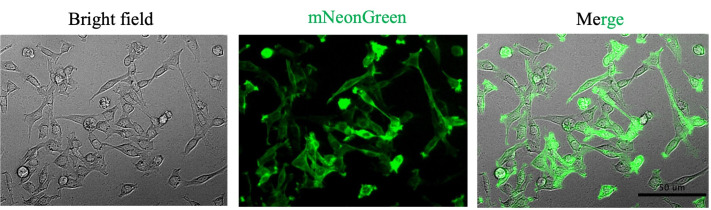
Representative images of mouse satellite cells expressing LifeAct-mNeonGreen, generated by retroviral transduction. The brightfield, mNeonGreen, and merged channels are shown here.

3. Aspirate the culture medium from the dish and add the virus-containing fresh GM. Return cells to the incubator.

4. After 24 h, aspirate the virus-containing medium, wash the dish gently with 1× PBS, and add fresh GM.

5. After an additional 24 h, satellite cells should exhibit robust expression of the transgene (e.g., LifeAct-mNeonGreen). *Note: Under the conditions described in this protocol, transduction efficiencies typically exceed 80% (Figure 8).*

## Validation of protocol

This protocol has been validated in the following research articles using multiple retroviral constructs, including pMx-LifeAct-mNeonGreen, pMx-LifeAct-mScarleti, pMx-Arp2-mNeonGreen, pMx-EGFP, and pMx-mScarleti to demonstrate its robustness and reproducibility:

• Lu et al. [27]. Spatiotemporal coordination of actin regulators generates invasive protrusions in cell–cell fusion. *Nature Cell Biology* (Extended Data Figure 1).

• Lu et al. [7]. Branched actin polymerization drives invasive protrusion formation to promote myoblast fusion during mouse skeletal muscle regeneration. *eLife* (Figures 3 and 4).

## General notes and troubleshooting

**General notes**

1. Freshly isolated satellite cells typically proliferate slowly; their proliferation rates increase with subsequent passages.

2. Because satellite cells are sensitive to serum levels, the virus should be concentrated after packaging and re-diluted in fresh GM for infection to maintain cells in a proliferative, undifferentiated state.

3. Retroviral transduction efficiency should exceed ~80% under optimal conditions. If higher purity is required, infected cells can be further enriched using a selection marker (e.g., puromycin) or by fluorescence-activated cell sorting.

**Troubleshooting**

**Problem 1:** Poor cell yield after satellite cell isolation.

Possible causes: Suboptimal mincing of muscle tissue can compromise dissociation efficiency. Over-mincing generates a dense suspension after initial collagenase digestion, preventing efficient pelleting of myofibers and leading to their loss in step A4. Conversely, under-minced tissue will not be fully digested and interferes with subsequent dissociation.

Solution: Cut muscle tissue into approximately 3-mm pieces.

**Problem 2:** Low satellite cell viability after thawing.

Possible causes: Cells are not cryopreserved or thawed properly.

Solutions: 1) Ensure that satellite cells are cryopreserved when they are healthy and actively proliferating. 2) Freeze cells slowly using an appropriate freezing medium (GM containing 10% DMSO); place the cryovials in a cell freezing container (e.g., CoolCell LX cell freezing container, Corning, catalog number: CLS432002). 3) Thaw cells rapidly in a 37 °C water bath. Immediately dilute the cells into prewarmed GM after thawing to minimize DMSO exposure.

**Problem 3:** Fibroblast overgrowth during satellite cell maintenance.

Possible causes: Fibrotic tissue is not sufficiently removed during muscle dissection, or pre-plating after isolation was insufficient to remove fibroblasts.

Solutions: 1) Carefully remove fibrotic and connective tissues during muscle dissection in section A2. Using a dissection microscope can be helpful for identifying and removing these tissues when necessary. 2) Extending the pre-plating time in section A4 to 2 h per round after isolation can further reduce fibroblast contamination, although this may decrease the yield of satellite cells due to increased satellite cell attachment during pre-plating.

**Problem 4:** Poor retroviral transduction efficiency.

Possible causes: Low viral titer or suboptimal proliferation of satellite cells.

Solutions: 1) Ensure that platinum-E cells are healthy and actively growing, as this is critical for efficient viral packaging and producing high-titer virus. 2) Ensure that satellite cells are highly proliferative at the time of infection, as retroviral infection requires cell division for efficient integration.
